# Fatal case of community-acquired pneumonia coinfected with monkeypox virus: clinical and autopsy findings

**DOI:** 10.1128/asmcr.00024-24

**Published:** 2024-12-10

**Authors:** Anselmo Abdo, Sergio Rabell, Fidel Simón, Zaddy Quintero, María C. de Armas, Namibia Espinosa, Lianet Quiles, Ileana Paneque, Karel Duran, Genny Martínez, Luis M. Pérez, Yenisey Pérez, Roberto Castellanos

**Affiliations:** 1 Center for Medical and Surgical Research, Havana, Cuba; 2 Legal Medicine Institute, Havana, Cuba; 3 Public Health Ministry, Havana, Cuba; Vanderbilt University Medical Center, Nashville, Tennessee, USA

**Keywords:** monkeypox, autopsy, hypoxic encephalopathy, pneumonia, community-acquired pneumonia

## Abstract

**Background:**

Monkeypox (Mpox) has been declared an international public health emergency. The Mpox infection is commonly auto-limited. However, complications, such as secondary bacterial infection, including pneumonitis and encephalitis, have been described, which can lead to death. We report the clinical evolution and autopsy findings of a patient with an atypical presentation and clinical course.

**Case Summary:**

The patient had no significant past medical history and was diagnosed with community-acquired pneumonia and monkeypox virus co-infection. The studied subject developed cardiorespiratory arrest with asystole and hypoxic encephalopathy, leading to death around 72 h after hospitalization. The main macroscopic findings in the lungs were condensation associated with bronchopneumonia in the lower regions of both lungs and not thrombi in the pulmonary artery. In the heart, no myocardial abnormalities and no evidence of valvular or vascular abnormalities were observed. Congestion was found in the kidneys and liver. Moderate to severe edema along with congestion of the leptomeninges was found in the brain. The main histopathological findings were lung tissue with diffuse lympho-histiocytic infiltrate, hemorrhagic necrosis of alveolar septa, vascular congestion, and intra-alveolar edema. The perivascular lymphocytic infiltrate in Virchow–Robin spaces was suggestive of encephalic abnormalities. Differentiation between complications due to Mpox and complications associated with Mpox was a limitation due to the lack of immunohistochemical and molecular studies in tissues.

**Conclusion:**

This case illustrates the severe Mpox disease that can be developed in immunocompetent, non-HIV individuals and the possible association of Mpox with other infectious diseases acquired in the community.

## INTRODUCTION

Monkeypox (Mpox) is a zoonosis caused by a virus of the genus Orthopoxvirus, family *Poxviridae*, that is reported mostly in Africa (1). On 23 July 2022, the General Director of the World Health Organization (WHO) declared the international outbreak of Mpox, which began in May 2022, to be an international public health emergency (2). In May 2023, after a sustained decrease in worldwide cases, it was declared to have ended. On 14 August 2024, the increase in Mpox cases in the Democratic Republic of the Congo and its spread to neighboring countries again led to a declaration of a Public Health Emergency of International Concern. Since 1 January 2022, Mpox cases have been reported to the WHO from 123 member states across all six WHO regions. As of 31 August 2024, a total of 106,310 laboratory confirmed cases and 0 probable cases, including 234 deaths, have been reported to WHO. The number of new cases reported monthly has increased by 15.6%, and the majority of cases were notified from the regions of Africa and the Americas (3).

The Mpox infection is commonly auto-limited. However, complications, such as secondary bacterial infection at sites of skin or mucosal lesions and extra-cutaneous disease, including pneumonitis and encephalitis, which can lead to death, have been described (4, 5).

Here, we report the clinical evolution and autopsy findings of a patient with an atypical presentation and clinical course. Our aim is to illustrate to the medical community the severe Mpox disease in an immunocompetent, non-HIV individual and its association with other infectious diseases acquired in the community.

## CASE PRESENTATION

A 50-year-old male, heterosexual, with no significant past medical history visited our emergency room 4 days after arriving from a European country with multiple active cases of Mpox. He was complaining of 3 days with asthenia, loss of appetite, fever, headache, odynophagia, and muscle aches. On physical examination, there were signs of dehydration. Unexpectedly, the patient developed cardio-respiratory arrest with asystole. The cardiopulmonary resuscitation (CPR) returned the patient to sinus rhythm without motor response or awakening and clusters of tonic–clonic seizures. Next, he was transferred to the intensive care unit with a blood pressure of 120/70 mmHg, a heart rate of 78 BPM (without amine support), and a temperature of 38°C. The patient was intubated and ventilated, with 92% SaO_2_ with a Glasgow coma scale (GCS) of 3. He also presented myotic pupils with a weak response to light, decreased osteotendinous reflexes, and no meningeal signs. Clinical ultrasound was performed, observing condensations in both lower lungs and adequate cardiac contractility without pericardial effusion.

**Fig 1:**
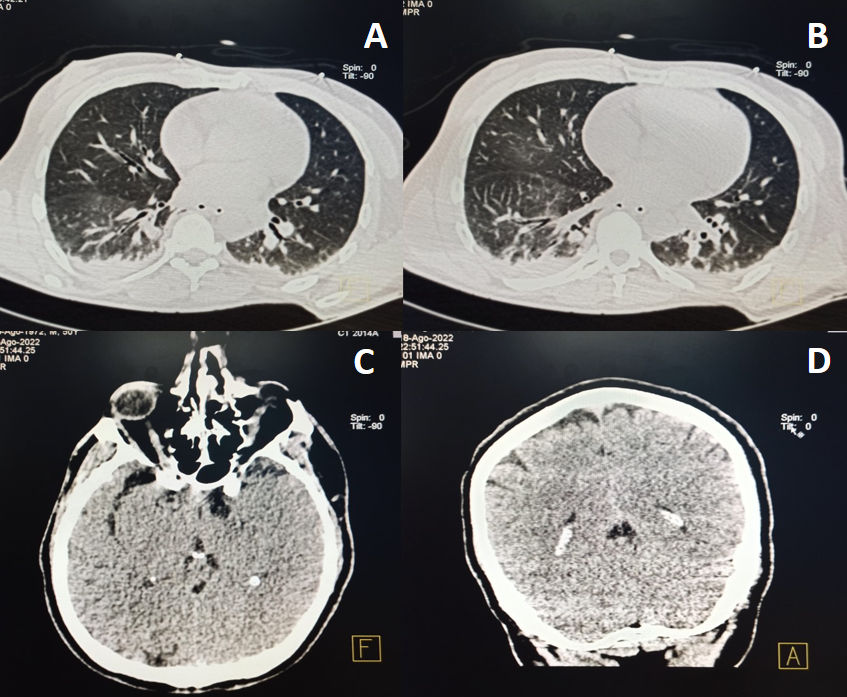
(A, B) Computed tomography (CT) of the chest with heterogeneous hypodense images with aerial bronchogram in both lungs more evident in the lower regions and related to an inflammatory process. (C, D) Cranial CT without cranio-encephalic lesions.

The electrocardiogram showed sinus rhythm without T or ST segment abnormalities. Chest and cranial computed tomographies were performed (Fig. 1). The clinical lung ultrasound and CT data indicated the presence of a community-acquired pneumonia (CAP). Furthermore, other tests suggested hypoxic encephalopathy related to cardiac arrest. Treatment with amoxicillin–sulbactam 1000/500 mg/8 h (iv) was initiated. Initial and follow-up blood work is shown in Table 1.

**TABLE 1:** Hematological tests from days 1 to 4 in the fatal Mpox case^*a*^

Test	Reference	Day 1 emergency room	Day 1 ICU	Day 2 ICU	Day 3 ICU	Day 4 ICU
Hemoglobin (g/dL)	13–17	N/D	11.6	13.8	13.8	12.4
Leukocytes (10^9^/L)	5–10	N/D	12.2	17.2	3.9	5.9
Neutrophils (%)	40–60	N/D	74	87	82	95
Lymphocytes (%)	20–40	N/D	17.2	4.3	7.6	3.3
Platelets (10^9^/L)	150–350	N/D	240	281	160	218
Prothrombin (s)	12–16	N/D	13/19	13/19	13/22	13/25
TGO (U/L)	10–40	N/D	23	69	93	107
TGP (U/L)	7–40	N/D	140	170	138	179
GGT (U/L)	5–40	N/D	N/D	35	23	15
Total bilirubin (μmol/L)	<19	N/D	N/D	10	15	17
Creatinine (mmol/L)	61.9–114.9	N/D	N/D	101	81	126
Urea (mmol/L)	2.1–8.5	N/D	N/D	7.7	7.7	13.8
Blood glucose (mmol/L)	4–5.6	6.5	10	12.1	9.2	8.7
CPK (U/L)	32–294	N/D	2262	3086	1932	N/D
CPK MB (U/L)	<6%	N/D	68	62	33	N/D
Troponin (ng/ml)	<40	N/D	<40	260	11	N/D
Procalcitonin (ng/mL)	<0.5	N/D	N/D	N/D	3.13	N/D
pH	7.35–7.45	6.96	7.32	7.22	7.37	7.38
PCO_2_ (mmHg)	38–42	72.7	36.3	54	46.6	50.4
PO_2_ (mmHg)	75–100	112.5	189.1	111.2	72.5	45.1
FiO_2_ (%)	21	21	50	40	40	40
EB (mmol/L)	±2.3	−15.8	−6.7	−6.6	1.1	3.6
HCO_3_ (mmol/L)	21–25	16.1	18.5	21.6	26.8	29.5
O_2_ saturation (%)	94–100	92.9	99.5	96.8	93.9	78.2
Na (mEq/L)	135–145	141.5	138.1	141.3	147.7	151
K (mEq/L)	3.7–5.2	3.3	4.1	4.7	3.86	4.2
Cl (mEq/L)	96–106	103	103.1	103.1	105.9	107.4
Ferritin (ng/mL)	12–300	N/D	N/D	N/D	N/D	228

^*a*^ ICU, intensive care unit; N/D, not done.

**Fig 2:**
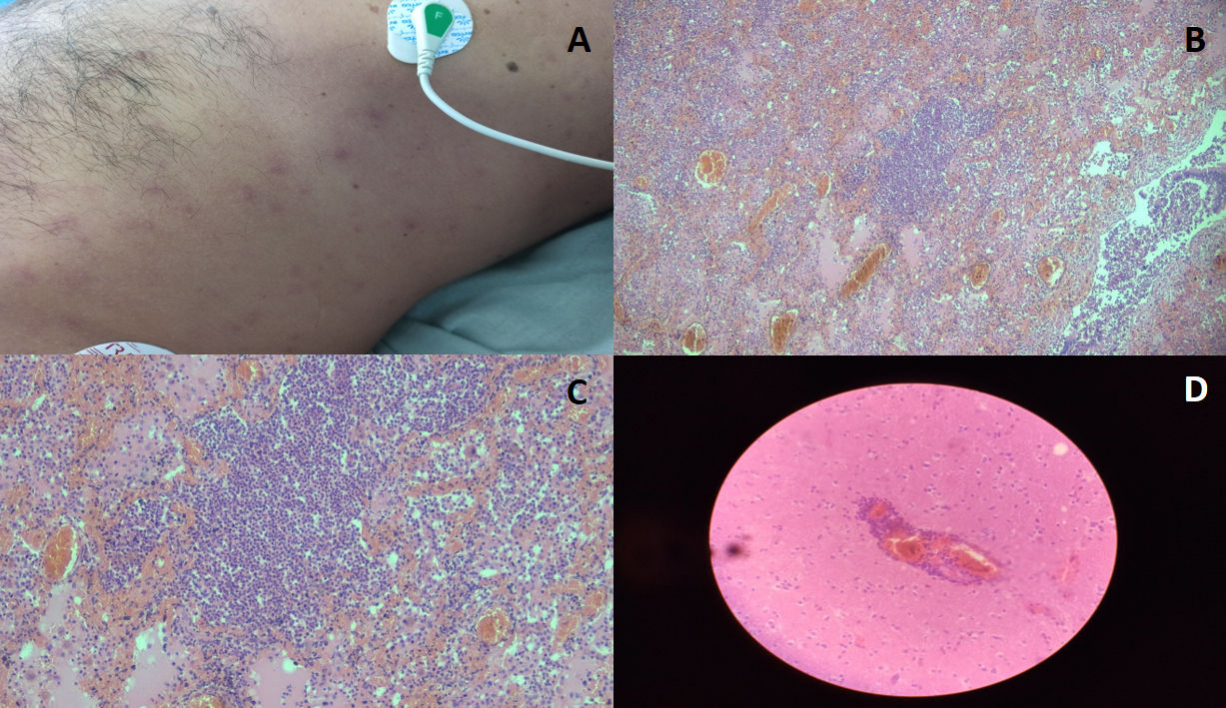
(A) Macular lesions on the trunk: some of them have small whitish papules in the center; (B) (×4) diffuse pulmonary damage; (C) (×10) lung tissue with diffuse lympho-histiocytic infiltrate, hemorrhagic necrosis of alveolar septa, vascular congestion, and intra-alveolar edema; and (D) (×40) perivascular lymphocytic infiltrate in Virchow–Robin spaces, suggestive of encephalic abnormalities. Staining used: hematoxylin and eosin.

On physical examination, macular lesions appeared in the trunk (Fig. 2, panel A). Skin samples were submitted to the National Reference Center for Virology and were positive for Mpox virus by qPCR with a Ct of 23. On the contrary, HIV antibody tests, venereal disease research laboratory test, severe acute respiratory syndrome coronavirus 2 (qPCR) test, and testing for dengue viruses (IgM Rapid Kit, IgG, NS1 Ag dengue, and PCR), varicella–zoster virus (qRT-PCR), and influenza A virus (qRT-PCR) all returned negative. Negative results were obtained from the toxicological analysis. On the fourth day, the subject maintained a temperature of 38.5°C and presented rales of the inferior lobes and respiratory secretions. Treatment with meropenem 2 g/8 h (iv) was also decided. Hemodynamic abnormalities required norepinephrine administration. After hemodynamic stabilization was achieved, the patient maintained a GCS of 3, along with no osteotendinous reflex, non-reactive mydriasis, corneal reflex, coughing reflex, and pharyngeal reflex. The atropine test did not modify the heart rate, while the apnea test showed the absence of respiratory effort. Before the second assessment, the patient presented cardiac arrest in asystole and did not respond to CPR. Death was pronounced after 72 h of hospitalization.

**Fig 3:**
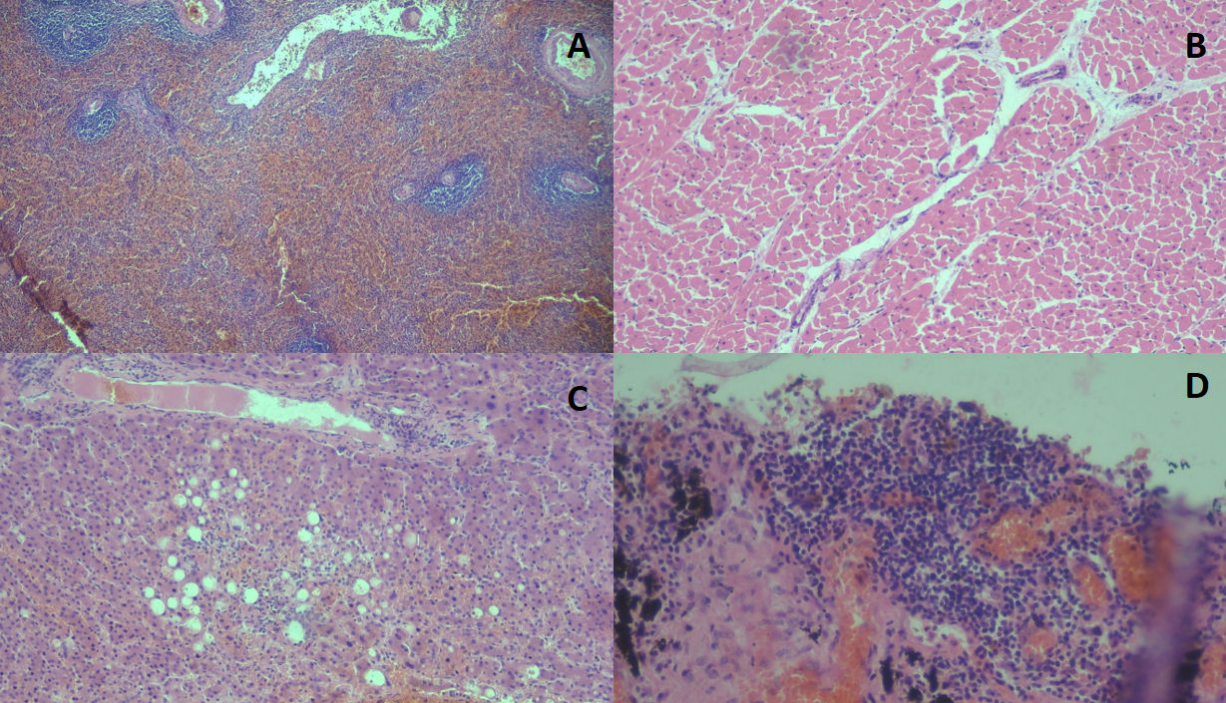
(A) (×4) Acute reactive splenitis; (B) (×10) myocardial tissue with no abnormalities; (C) (×10) microvacuolar hepatic steatosis; and (D) (×10) lung tissue with lymphomonocyte infiltrate and pulmonary anthracosis. Staining used: hematoxylin and eosin.

Macroscopic autopsy findings in the pulmonary system were lung condensation associated with bronchopneumonia in the lower regions of both lungs and not thrombi in the pulmonary artery. In the heart, no myocardial abnormalities and no evidence of valvular or vascular abnormalities were observed. Congestion was found in the kidneys and liver. Moderate to severe edema along with congestion of the leptomeninges was found in the brain. The histopathological analysis is shown in Fig. 2B through D and Fig. 3.

At the time of hospitalization at the intensive care unit, the blood culture revealed no bacterial growth. No evidence of autochthonous transmission of the virus was found after more than 21 days of contact surveillance.

## DISCUSSION

The fatal clinical evolution of Mpox is not common. During the initial outbreaks of the disease in Africa and the outbreak in the United States, the fatal cases reported were children (6, 7). However, in the current international emergency, most fatalities have been men with an HIV diagnosis and who have sex with men (8).

Jezek et al. (6), in a clinical characterization of 282 patients diagnosed with Mpox in Zaire from 1980 to 1985, reported the deaths of 12 patients. All the fatal cases were children between 3 months and 8 years of age. On the contrary, Huhn et al. (7), in 34 patients who became ill with Mpox in the US outbreak in 2003, did not report fatal adult cases but two pediatric patients requiring intensive care, that is, a 6-year-old girl with encephalitis and a 10-year-old girl with respiratory distress.

In the current outbreak, Rodrigues-Meneces et al. (4) described a Brazilian fatal case with no autopsy. The patient was HIV-positive with a lymphoma and died from multiple organ failure secondary to sepsis. Secondary bacterial infections in skin and mucosal lesions, as well as respiratory infections, have been the main causes of death from Mpox. Ritter et al. (9) reported histopathologic findings and coinfections found in the tissues of 16 severe Mpox patients and six fatal Mpox patients. All patients were severely immunocompromised, with 20 having HIV/AIDS and two patients being renal transplant recipients receiving immunosuppressant drugs. Coinfections were identified in specimens from 5 of 16 (31%) biopsy cases and in 4 of 6 (67%) autopsy cases. Among the autopsy patients, CMV was detected in three patients, and two of those patients developed polymicrobial pneumonia—one case with non-pneumococcal *Streptococcus* spp. and human parainfluenza virus-3 and another with *Pneumocystis* spp. and mixed bacteria, including *Escherichia coli* and *Staphylococcus* spp. In another autopsy patient, Gram-negative bacterial pneumonia, CMV, and *Candida* spp. infections were identified.

Riser et al. reported 38 fatalities due to Mpox in the United States. HIV infection was present in 93.9% of cases, and the average time between admission and death was 68 days. The work did not discuss the organ failure that led to death (10).

Autopsy results from a fatal Mpox case with a history of HIV infection revealed septic shock secondary to respiratory infection and Mpox-positive PCR in tissue samples from brain, bone marrow, and testes (11).

Fink et al. described the evolution of 156 patients admitted to 16 U.K. hospitals with Mpox complications (12). Secondary bacterial infections in the skin lesions were reported in 58% of patients. Two cases developed encephalitis by Mpox, and the orthopox DNA PCR was positive in the cerebrospinal fluid. Both patients recovered, and no fatalities were reported in this study (12). Mpox-related myocarditis was not common, with six non-fatal cases reported in 2022 (13).

In the present case, we believe that the direct cause of death was hypoxic encephalopathy secondary to cardiac arrest in an individual with CAP coinfected with Mpox. The initial symptoms, radiological studies, and histopathological findings were suggestive of CAP possibly initiated by a viral infection or other common respiratory microorganisms that can explain the type of pulmonary inflammatory infiltrate (*Mycoplasma pneumoniae* or *Chlamydia pneumoniae*). Our work has a diagnostic limitation. No Mpox immunohistochemical and molecular studies were performed in tissues from internal organs, which prevented a clear differentiation between complications due to Mpox and those associated with Mpox. In the studied case, it seems more likely the latter hypothesis.

In conclusion, we report herein an Mpox case in a non-HIV-positive individual associated with CAP that evolved to death in a short period of time. The evolution and outcome were atypical in comparison with most of the reported Mpox fatalities.
